# Olive Oil Polyphenols: A Promising Approach for Cancer Prevention and Therapy

**DOI:** 10.1002/fsn3.70976

**Published:** 2025-09-18

**Authors:** Muhammad Junaid Anwar, Muhammad Hammad Anwar, Muhammad Imran, Ahmad Mujtaba Noman, Muzzamal Hussain, Hassan Raza, Hagar M. Mohamed, Gamal A. Mohamed, Sabrin R. M. Ibrahim, Tadesse Fenta Yehuala, Suliman A. Alsagaby, Waleed Al Abdulmonem, Mohamed A. Abdelgawad, Ehab M. Mostafa, Mohammed M. Ghoneim, Samy Selim

**Affiliations:** ^1^ Department of Food Science and Technology, Faculty of Food Science and Nutrition Bahauddin Zakariya University Multan Pakistan; ^2^ Department of Food Science and Technology, Faculty of Food and Home Science Muhammad Nawaz Sharif University of Agriculture Multan Multan Pakistan; ^3^ Department of Food Science and Technology University of Narowal Narowal Pakistan; ^4^ Department of Human Nutrition, Faculty of Food Science and Nutrition Bahauddin Zakariya University Multan Pakistan; ^5^ Department of Food Sciences Government College University Faisalabad Faisalabad Pakistan; ^6^ Department of Medical Laboratory Analysis, College of Medical & Health Sciences Liwa University Abu Dhabi UAE; ^7^ Department of Applied Medical Chemistry, Medical Research Institute Alexandria University Alexandria Egypt; ^8^ Department of Natural Products and Alternative Medicine, Faculty of Pharmacy King Abdulaziz University Jeddah Saudi Arabia; ^9^ Department of Chemistry, Preparatory Year Program Batterjee Medical College Jeddah Saudi Arabia; ^10^ Department of Pharmacognosy, Faculty of Pharmacy Assiut University Assiut Egypt; ^11^ Faculty of Chemical and Food Engineering, Bahir Dar Institute of Technology Bahir Dar University Bahir Dar City Ethiopia; ^12^ Department of Medical Laboratory Sciences, College of Applied Medical Sciences Majmaah University AL‐Majmaah Saudi Arabia; ^13^ Department of Pathology, College of Medicine Qassim University Buraidah Kingdom of Saudi Arabia; ^14^ Department of Pharmaceutical Chemistry, College of Pharmacy Jouf University Sakaka Saudi Arabia; ^15^ Department of Pharmacognosy, College of Pharmacy Jouf University Sakaka Saudi Arabia; ^16^ Department of Pharmacy Practice, College of Pharmacy AlMaarefa University Ad Diriyah Saudi Arabia; ^17^ Department of Clinical Laboratory Sciences, College of Applied Medical Sciences Jouf University Sakaka Saudi Arabia

**Keywords:** hydroxytyrosol, lung cancer, oleuropein breast cancer, olive oil, polyphenols, STAT3

## Abstract

Cancer is emerging as a leading cause of death globally. Considering the disease burden, scientists are working to develop various strategies by exploring natural sources for cancer treatment. The use of olive oil (OO) and its phytochemicals, specifically oleuropein and hydroxytyrosol (HT), as a remedy against various types of cancer is gaining attention among scientists and researchers. The current review aimed to highlight the antioxidant potential and summarize the various pathways and markers involved in the prevention and treatment of different human cancers. The databases, including Google Scholar, PubMed, and Web of Science, were searched to collect relevant data. The studies showed that some markers are specifically linked to particular types of cancers (such as 27‐OHC, ATF‐2, TNFRSF10B, HER2, and MET, which are linked to breast cancer; iNOS and NO, which are linked to thyroid cancer; GLUT1 and GLUT4, HIF‐1α, MCT4, PKM2, PD‐1, PD‐L1, and CTLA‐4, which are linked to colorectal cancer). Olive oil is involved in the management of these markers to prevent and/or treat all types of cancers by upregulating tumor suppressor genes, downregulating oncogenes, and modulating different pathways (PI3K/AKT/mTOR, Wnt/β‐catenin, and MAPK). Taken together, it can be concluded that olive oil and its components have prominent potential to cure cancers in the human body. However, further investigations can be carried out to suggest the lethal doses of olive oils as a whole and its phytochemicals individually for the treatment of cancer.

## Graphical abstract

The manuscript explores the anticancer potential of olive oil polyphenols, specifically hydroxytyrosol and oleuropein, which exhibit potent antioxidant, anti‐inflammatory, and anticancer properties. These bioactive compounds modulate key molecular pathways involved in the initiation and metastasis of cancer. They induce apoptosis, inhibit angiogenesis, and suppress the proliferation of tumor cells. Their natural origin and multiple mechanisms of action make them promising candidates for integrative cancer therapy. However, clinical trials are required to validate their anticancer effects and further pharmaceutical applications.

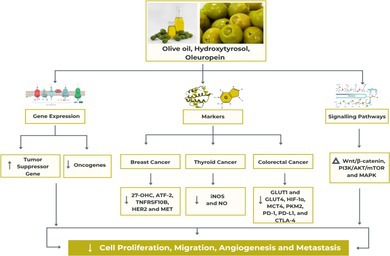

## 1 Introduction

A compelling trend has emerged in the development of pharmaceuticals since the late 1990s, with a return to nature as a potential source of drugs (Xiao et al. 2017; Xiao 2017). Medicinal plants such as myrobalan, olive, and several other plants are being used to treat various disorders, including cancers, cardiovascular diseases, diabetes, and hypertensive disorders, as a natural medicine (Sultan et al. 2023). Olive oil has gained importance since ancient times due to its particular therapeutic properties and health‐promoting attributes. Olive (*Olea europaea L*.), belonging to the plant Oleaceae family, is an evergreen shrub which matures to produce olive fruit. The shrub can grow as high as 15 m and slowly matures, but it can survive for several decades (Martakos et al. 2021). Its origin is based in the Mediterranean basin area and some parts of Asia Minor. However, it has a worldwide cultivation, particularly in Mediterranean areas, regions of the Asia‐Pacific, and also in South and North America (Russo et al. 2020). Throughout the Mediterranean area's civilization history, leaves of the olive were utilized for folk medicine, while its fruit was served as table olives. However, the primary olive plant product is the oil, which is valued globally for its distinctive nutrients and flavor. Homer named it the “liquid gold” (Lorini et al. 2021).

The composition of olive oil comprises a complex array of more than 200 compounds, including both minor and major, yet indispensable ones (Aiello et al. 2015). Olive oil's chemical composition consists mainly of the polyunsaturated fatty acids, monounsaturated fatty acids, and the saturated fatty acids, primarily in glycerol esters. Moreover, olive oil also contains reasonable amounts of vitamins and minerals, including vitamin K and E, sodium, calcium, potassium, and iron (Boskou 2015). Additionally, olive oil's bioactive components are divided into the following two categories: (1) nonpolar (triterpene, sterols, tocopherols along with squalene compounds); (2) polar (termed as phenolic compounds as the “polyphenols”). Primarily, the major phenolic compounds, such as hydroxytyrosol and its derivatives (tyrosol and oleuropein), along with the rich nutritional composition of olive oil, provide various health benefits and therapeutic attributes, including antioxidant, anti‐inflammatory, anticancer, antidiabetic, and cardioprotective effects (Nowak et al. 2021).

Morbidities and mortalities due to cancer are growing rapidly worldwide, which demands a cost‐effective, natural, and safe approach to reduce the prevailing burden (Noman et al. 2025). In the developed Western countries, the new cancer numbers are increasing, which are related to the exposure to environmental risks and unhealthy lifestyles (Bray et al. 2018). However, lower cancer incidence has been observed in Mediterranean regions, associated with dietary factors, compared to European countries or the USA. The olive oil and its polyphenolic compounds, which exhibit anticancer and antitumoral activities, have been widely studied in both in vitro and in vivo studies (Reboredo‐Rodríguez et al. 2018). Evidence from different studies suggests that the intake of olive oil is inversely linked with the risk of any cancer type (van den Brandt and Schulpen 2017).

This review stands out for comprehensively exploring the chemopreventive and therapeutic potential of olive oil polyphenols, particularly hydroxytyrosol and oleuropein, in various types of cancer. It uniquely integrates molecular mechanisms, including antioxidant, anti‐inflammatory, antiproliferative, and proapoptotic pathways, supported by both in vitro and in vivo studies. The strength lies in its multidisciplinary approach, linking nutritional science with oncology. By emphasizing the role of diet‐derived bioactive compounds in cancer modulation, the review contributes novel insights into functional foods, making it valuable for both researchers and healthcare professionals. Table 1 describes the role of oleuropein in the treatment of different cancer types.

**TABLE 1:** Role of oleuropein in the treatment of different cancer types.

Types of cancer	(In vitro/In vivo/Ex vivo)	Cell lines/Model	Dose	Mode of action/Effects	References
Breast	In vitro	MDA‐MB‐231 and MCF‐7 cell line	1000 μL of extra virgin olive oil	Reduce proptosis, reduced SOD, inhibit PAI‐1	Tzekaki et al. (2021); Arı et al. (2018)
Cervical	In vitro	GH329 and mice	—	miR‐499a indorse the propagation, cell cycle progress, invasion, miR‐499a aimed SOX6 and mediated oncomirs effects	Chen et al. (2020)
Colorectal	In vivo	C57BL/6 mice	50 and 100 mg/kg of oleuropein	Limitation in Th17 response, decrease in IL‐6, IL‐17A, IFN‐γ, and TNF‐α, reduction in Wnt/β‐catenin, cyclooxygenase‐2 and Bax, STAT3 dysregulation, NF‐κB, and P3IK/Akt	Giner et al. (2016)
Gastric	In vivo and in vitro	SGC‐7901, AGS cells and MGC‐803	—	Induced apoptosis, High KRAS, low miR‐200; miR‐760 high, apoptosis enhancement, and reduction in MMP role in MGC‐803 cell lines	Zhou et al. (2020); Barzegar et al. (2019)
Ovarian	In vitro	Follicles of ovary, thecal cell	1% (w/v) Oleuropein drinking water	Elevation of HPSE1, reduction miR‐299, amplified sensitivity to radiation	Xing et al. (2017)
Prostate	In vitro & in vivo	mice, LNCaP, DU145, PC3, and MAT‐LyLu cell	10, 25, 50, 100 mM concentrations of aqueous olive leaf extract (AOLE)	Hindering voltage‐gated sodium routes; antimetastatic miR‐381 high, stimulating PCa cell death and autophagy	Aktas and Ayan (2021)
Thyroid	In vitro	BCPAP cell and TPC‐1	10, 25, 50, 100 μM concentrations of aqueous oleuropein and per‐acetylated oleuropein	Reduced p‐Akt and p‐ERK; inhibit propagation	Bulotta et al. (2013)
Colon	In vitro	HT29 cells	Oleuropein and hydroxytyrosol in 2 mL of serum complete media	Influences IκBα, regulating the NF‐κB canonical pathway, suppresses COX‐2 expression and the Wnt/β‐catenin signaling pathway, inhibits the p38 signaling pathway and transcription factor CREB, modulates PPARγ levels.	Cárdeno et al. (2013)
Seminoma	In vitro	TCAM‐2 and SEM‐1	Oleuropein 15–200 μM for 48 h	BAX overexpression	Bossio et al. (2022)
Breast	In vitro	MIDA‐MB‐468	500 μM in MDA‐MB‐231 cells and 250 μM in MDA‐MB‐468 cells for DNA extraction in TRIzol	Enhancing the expression of multiple caspases, including caspases 1 and 14.	Messeha et al. (2020)

## 2 Olive Oil Polyphenols Mechanism Linked With Amelioration of Cancer

Bioactive compounds have the potential to modulate key molecular pathways in cancer, including apoptosis, cell cycle arrest, angiogenesis, and metastasis. Primarily, they influence signaling pathways such as PI3K/Akt, STAT3, MAPK, and NF‐κB, suppress oncogene expression, and enhance the expression of tumor suppressor genes. Thus, these compounds offer promising therapeutic potential in cancer prevention and treatment (Maaz et al. 2025).

### 2.1 Modulation of STAT3 Pathway

Studies have determined the association of STAT3 with cell proliferation and metastasis. Therefore, the mutation in STAT3 results in malignancy and tumorigenesis. It is activated by phosphorylation due to cytokines and other growth factors, binds to DNA, activates gene transcription, and initiates cell proliferation. STAT3 is involved in inflammation, including the NF‐κB and IL‐6 pathways (Hu et al. 2024). Shi et al. (2020) investigated the role of STAT3 and OLP (50 and 100 mg/kg) in keratinocyte proliferation. They demonstrated that OLP reduced psoriasis skin lesions by ameliorating IL‐17, improving infiltration of CD3+, CD4+, and CD8+ T cells, blocking the differentiation of Th17, and modulating the JAK3/STAT3 pathway. Another study demonstrated that OLP (20 mg/kg) inhibited TNF‐α, IL‐1β, and IL‐6 levels, while also improving SOD, GSH, and CAT, and modulating the MEK/ERK/STAT3 signaling pathway. Gu et al. (2017) reported that oleocanthal (0–60 μM) suppressed proliferation and angiogenesis in A375, A2058, HUVEC, and HaCaT cell lines by downregulating STAT3 target genes, including Mcl‐1, Bcl‐xL, MMP‐2, MMP‐9, and VEGF, and inhibited the expression of Ki‐67 and CD31.

Moreover, STAT3 is involved in the development and progression of hepatocellular carcinoma (HCC) and colorectal cancer (CRC). Besides STAT3, the overexpression of SMYD2 and c‐MET is responsible for CRC. Tarun et al. (2025) investigated the impact of oleocanthal (OC) against CRC in a nude mouse xenografting model and HCT‐116, COLO‐320DM, and SW48 CRC cell lines. They reported that 10 mg/kg OC for 15 days inhibited 72.5% of the *KRAS* mutant HCT‐116‐Luc cell tumors. Moreover, expressions of SMYD2‐EZH2 and c‐MET activation were remarkably suppressed. Fogli et al. (2016) studied a group of mice treated with oleocanthal, which had smaller and fewer metastases in the lungs compared with the control group. Oleocanthal inhibited the metastasis and tumor growth of hepatocellular carcinoma (HCC) by deactivating STAT3 in both in vivo and in vitro studies. Hence, STAT3 can be a promising target for the therapy of HCC.

### 2.2 Immune Checkpoint Inhibition and PI3K/AKT Pathway

Tumor immune escape through the activation of immune checkpoint proteins, T‐cell exhaustion, decreased DNA repair, and metabolic reprogramming is critical for CRC initiation and progression. Programmed cell death 1 (PD1), PD‐L1, and CTLA4 are immune checkpoint proteins acting as inhibitory immune receptors (Tanjak et al. 2025). Concerning this, Ajayi and Adeshina (2023) studied the impact of EVOO (2 mL/Kg) on CRC and found that EVOO treatment increased the expression of *MSH2*, *PMS2*, *MLH1*, *P53*, *MSH6*, CD4+, and CD8+ T cells and reduced the expression of CTLA4, PD1, and PD‐L1. Furthermore, colonic expression of GLUT1 and GLUT4, HIF‐1α, lactate dehydrogenase, MCT4, and PKM2 was also suppressed in CRC mice. Oleuropein (OLP) (200 μg/mL) and its hydrolysate, comprising oleocanthal (3 μg/mL) and hydroxytyrosol (30 μg/mL), from EVOO, inhibit the proliferation of MCF‐7 and MDA‐MB‐231 breast cancer cells by modulating the PI3K/AKT signaling pathway. Moreover, these compounds induced cell apoptosis, cell cycle arrest at the G1/S phase, and reduced Cyclin D protein expression (Han et al. 2023; Gorzynik‐Debicka et al. 2018).

### 2.3 Cell Cycle Arrest and AKT/mTOR Phosphorylation Reduction

Oleuropein (500 μM) and oleocanthal (250 μM) also inhibited the proliferation of MDA‐MB‐231 and MDA‐MB‐468 cells. The results demonstrated that oleocanthal had a more substantial impact than oleuropein, and RNA sequencing exhibited that the expression profile of TNBC cells was substantially transformed after treatment with these compounds. They concluded that downregulation of matrix metalloproteinases (MMPs), cell cycle arrest at the S phase, upregulation of p21 expression, and reduction in phosphorylated mTOR were observed (Karousi et al. 2023). Furthermore, another study depicted that oleuropein, derived from the crude extracts of olive leaf, was used as a striking compound that reduced the proliferation of MCF‐7 cells, carcinoma of the human urinary bladder, and capillary endothelial cells of bovine brain. The in vitro study indicated that oleuropein possesses anticancer properties and decreases the growth and invasiveness of MCF‐7 cells in the lung. Oleuropein played a prominent role in preventing parenchyma and peripulmonary lung metastases (Sepporta et al. 2014).

**FIGURE 1:**
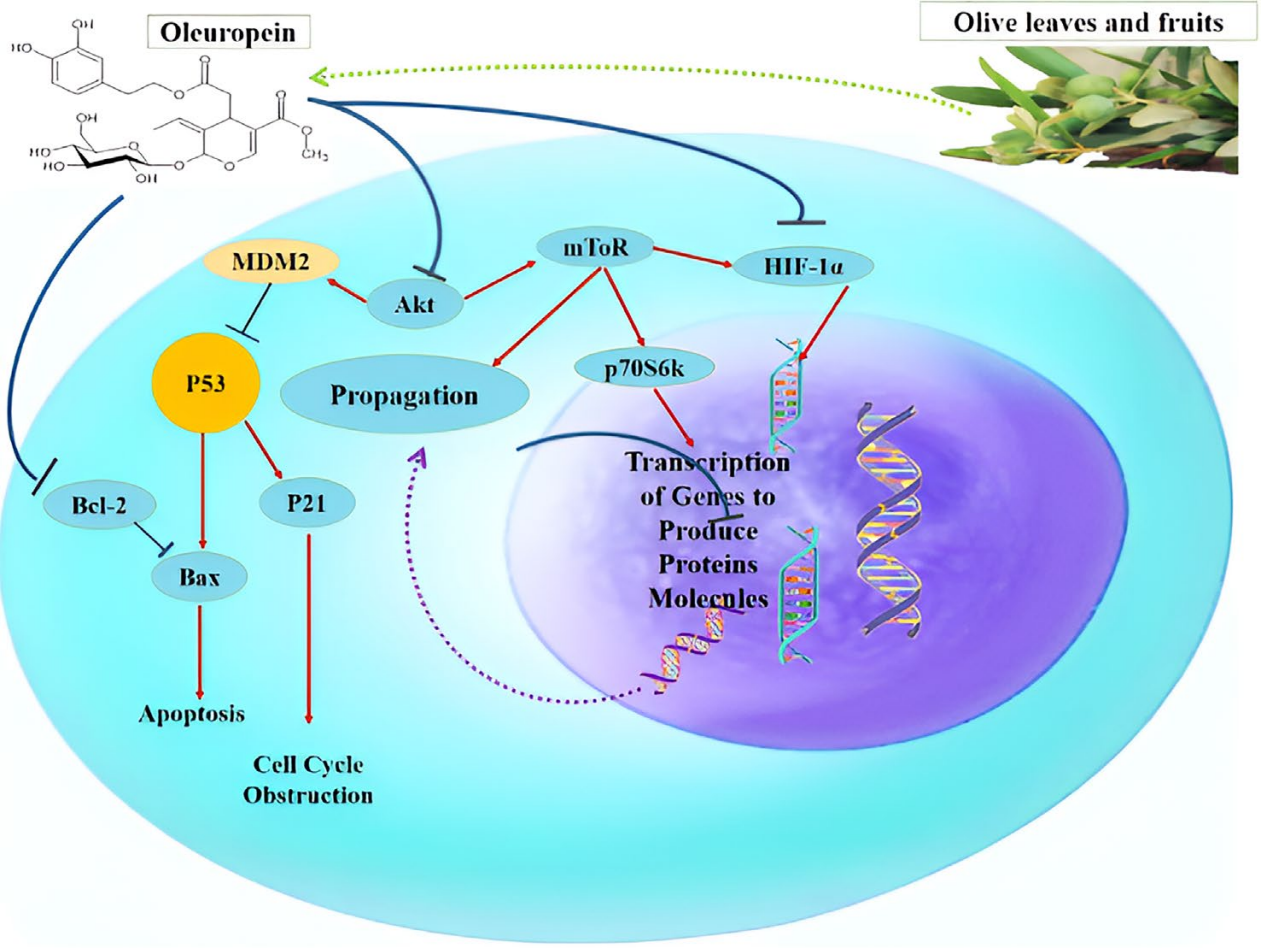
Depicting the mode of action of oleuropein against cancer.

Recent research has indicated that oleocanthal minimizes the extracellular signal‐regulated kinases (ERK1/2) along with AKT phosphorylation and downregulates the expression of Bcl‐2. ERK1/2 and AKT are important for cell mortality, invasion, and adhesion, which are regulated by c‐Met‐mediated signaling. However, oleocanthal also controls mitogenesis and cell survival and induces cytotoxicity against human melanoma (Siddique et al. 2020). The meta‐analysis depicted that consumption of MED is linked to a 14% lower risk of colorectal cancer development. Moreover, it was also suggested that the antitumoral activity of EVOO lowered the incidence of colorectal cancer tumors in rats due to epigenetic mechanisms, specifically DNA and miRNA methylation (López‐Biedma et al. 2016). Figure 1 depicts the general mechanism of the potential mode of action of oleuropein against cancer.

### 2.4 Mechanism of Action of Olive Polyphenols by Using Bioinformatic Tools

Recent advances in bioinformatics provided valuable insights into the molecular mechanisms underlying the health benefits of olive polyphenols. Through an integrated computational approach, several platforms were employed to predict molecular targets, evaluate pharmacokinetic properties, and map regulatory networks. For instance, PubChem and SwissTargetPrediction were used for compound characterization and target identification, while SwissADME facilitated the assessment of drug‐like behavior and absorption, distribution, metabolism, and excretion (ADME) profiles. Furthermore, eXpression2Kinases (X2K) analysis revealed complex gene networks involving transcription factors and kinases. Collectively, these tools identified 29 human protein targets for key olive oil compounds, highlighting peroxisome proliferator‐activated receptor (PPAR) signaling as a central mechanism. Additional mechanistic insights suggested FABP‐mediated nuclear entry of fatty acids, multi‐PPAR activation conferring anti‐inflammatory effects, tyrosol‐mediated NF‐κB inhibition, and melatonin receptor signaling in circadian regulation. These findings emphasized that olive polyphenols acted through multiple interconnected pathways, thereby explaining their diverse physiological effects and therapeutic potential (Fatoki et al. 2021). In another network‐based bioinformatics study on oleuropein, researchers employed a systematic cheminformatics and pathway‐mapping approach. The compound structure was first retrieved from ChEBI and DIGEP‐Pred and then used to predict drug‐induced gene expression changes. The predicted genes were curated via GeneCards and subsequently integrated into a protein–protein interaction (PPI) network using STRING. Enrichment analysis through KEGG pathways revealed significant associations with TNF, IL‐17, PI3K–Akt, NF‐κB, and cancer‐related signaling cascades (Gezici and Sekeroglu 2021). Another comprehensive nutrigenomics investigation used multiple bioinformatics platforms to understand how olive polyphenols influence cardiometabolic health. GeneTrail2 was applied for pathway enrichment, while Cytoscape with ClueGO/CluePedia enabled functional clustering of gene groups. STRING identified high‐confidence PPI modules, and transcription factor prediction was carried out through Enrichr (TRRUST). For miRNA analysis, the study utilized miRBase in combination with TargetMiner, TargetScanVert, and miRDB, with InteractiVenn and Metascape employed for data integration and visualization. Network relationships were further explored using OmicsNet and presented in a three‐dimensional mRNA–TF–miRNA interaction view. Together, these analyses highlighted interconnected mechanisms involving PPAR signaling, inflammation, lipid metabolism, and endothelial function, underscoring that olive polyphenols act through complex regulatory networks rather than single targets (Ruskovska et al. 2021).

## 3 Antioxidant Potential of Olive Oil Polyphenols

The health advantages associated with EVOO are linked to its chemical structure and the presence of bioactive components that possess antioxidant potential. Phenolic acids function as antioxidants through the donation of electrons or hydrogen, whereas carotenoids serve as an antioxidant agent by decreasing singlet oxygen and free radicals (Slama et al. 2020). Secoiridoids are the primary classification of phenolic components found in EVOO, which are predominantly composed of derivatives of hydroxytyrosol and tyrosol. Minor amounts of lignin are also present, with pinoresinol and acetoxypinoresinol being the predominant ones. EVOO contains various phenolic acids, including coumaric, protocatechuic, and vanillic acids. Ultimately, certain flavonoids, specifically apigenin and luteolin, hold significant importance (Gorzynik‐Debicka et al. 2018). Both VOO and EVOO contain substantial quantities of alcoholic phenols, predominantly hydroxytyrosol and secoiridoids. Phenolic components exhibit a diverse range of biological activities, including the slowdown of disease progression associated with oxidative stress, owing to their antioxidant properties (Romani et al. 2019). Hydroxytyrosol is a well‐researched compound recognized for its potent antioxidant properties. It has been found to be nontoxic and without adverse effects up to a dosage of 500 mg/kg/day, as evidenced by the absence of a no‐observed adverse effect level (Rodríguez‐López et al. 2020).

According to Borges et al. (2019), the presence of antioxidants in EVOO can effectively delay the initial stages of oxidative stress through various mechanisms, including the prevention of oxygen free radical formation and propagation. The impact of various agronomic influences, that is, varieties, fruit ripening stage, and agroclimatic conditions, on the antioxidant properties and composition of EVOO is a well‐established phenomenon. The phenolic profile of extra‐virgin olive oil is primarily affected by the ripeness of the olives and the cultivar used. On the other hand, the flavor of the oil is more closely associated with climatic and geographic factors rather than the ripening stage and cultivar (Quintero‐Flórez et al. 2018).

The impact of maturity indices of olive (2.5, 3.5, and 4.5) on the antioxidant potencies and phenolic components derived from cultivar, Nizip Yaglik olives, was investigated by Amanpour et al. (2019). The predominant phenolic class observed in all samples was secoiridoids, with a notable decrease in their overall content as the olive fruits underwent ripening. The results indicate a noticeable decrease in antioxidant potency values as the olives mature. Negro et al. (2019) assessed the various phenolic profiles along with antioxidant capacities of mono varietal oils. The findings validated that the entire structure of oleuropein exhibits a fluctuation of up to fourfold among distinct genotypes, ranging between 33.80 and 152.32 mg/kg oil, which may be due to genetic variation, environmental conditions, agronomic practices, fruit maturity, and extraction methods (Lechhab et al. 2022). Zorić et al. (2021) explored the potential antioxidant and protective properties of phenolic components (hydroxytyrosol and oleuropein), found in the leaves and oil of the olive plant, against DNA damage generated by H_2_O_2_ in human peripheral lymphocytes. The pre‐exposure of lymphocytes to each of the compounds for 120 min resulted in a dose‐dependent decrease in primary DNA damage in the cell type. Hydroxytyrosol exhibited superior protection against H_2_O_2_‐induced DNA damage compared to oleuropein at the tested concentrations of 1, 5, and 10 μmol L^−1^. Wani et al. (2021) reported substantial changes in the antioxidant properties and phenolic compounds based on the cultivar, extraction conditions, and solvent. The submerged treatment of methanolic and ethanolic extracts resulted in a normal increase in their antioxidant properties, whereas submerged water extraction led to a significant decrease in the same.

In addition, Olajide et al. (2020) synthesized two lipophilic derivatives of hydroxytyrosol (HT), using 3,4‐dihydroxy phenyl acetic acid as the initial compound. The study's findings indicate that tert‐butyl‐hydroquinone demonstrated superior antioxidant properties in bulk oil at a temperature of 65°C compared to the novel HT derivatives. However, its efficacy was significantly reduced at higher temperatures exceeding 110°C. The results indicate that incorporating a voluminous alkyl group into the ortho‐diphenolic framework of HT led to an increase in its antioxidant efficacy. Serra et al. (2018) conducted a study on digestive disorders and found that the application of phenolic extracts from olive oil in vitro can mitigate the impacts of oxidized lipids, which can cause inflammation and oxidation, including oxysterols and hydroperoxides, on Caco‐2 cells, which are similar to enterocytes. The study demonstrated that the phenolic components present in olive oil exhibit a mitigating influence on the mitogen‐activated protein kinase/nuclear factor kappa B pathway. This pathway has been identified as a causative influencer in the synthesis of inflammatory bowel diseases. Incani et al. (2016) discovered that phenolics found in olive oil have a protective effect by inhibiting the formation of oxidative substances, controlling inflammatory mediators, and reducing the toxic effects of oxygenated cholesterol products found in cholesterol‐containing foods.

The study conducted by Fusco et al. (2020) aimed to assess the impact of HT administration on the injury caused by caerulein administration in the pancreas and intestine. The administration of HT treatment at a dosage of 5 mg/kg was initiated 30 min after the initial caerulein injection and continued for a duration of two consecutive hours. The study's findings indicate that HT was effective in reducing lipid peroxidation and oxidative stress in injured tissues. This was achieved through the enhancement of nuclear factor erythroid 2‐related factor 2 and heme‐oxygenase 1, which in turn resulted in increased activity of glutathione S‐transferase, glutathione reductase, glutathione peroxidase, and superoxide dismutase. Furthermore, a study conducted by Wu et al. (2018) observed that HT had a positive influence on preserving intestinal barrier integrity. This was demonstrated through the measurement of diamine oxidase levels in serum and the expression of constricted occludin and zonula occludens (junction proteins) in the colon and pancreas.

## 4 Effect of Olive Oil on Different Cancer Types

### 4.1 Brain Cancer

The anticancer effects of olive oil natural components involve various molecular pathways. Results from multiple research studies reveal that olive oil triterpenoids also act upon three different stages of carcinogenesis, including tumor initiation, promotion, and progression, by altering tumor behavior and various pathways (Gonçalves et al. 2024; Abd El‐Hafeez et al. 2018). The fundamental molecular markers that are known and validated prognostic variables include hormone receptors, such as estrogen, human epidermal growth factor receptor‐2 (HER‐2), progesterone receptor (PR), and estrogen receptor (ER). Together with clinicopathological prognostic factors, these molecular markers provide the most accurate prognostic information regarding cancer progression and recurrence (Li, Doherty, et al. 2022; Li, Cao, et al. 2022; Mohanty et al. 2022). According to a study, active sites, that is, N‐9, C‐1, and C‐3 of the norharmane nucleus were utilized to create novel compounds, and according to structure–activity relationship (SAR), those locations have been linked to anticancer properties which can be helpful to understand the mechanism of olive oil (Uthirapathy 2024; Sahoo et al. 2019). Olive oil's proapoptotic effects, induced by oleanolic acid and maslinic acid, were also illustrated in the tumors of malignant astrocytic tumors, which are a common primary brain tumor. It was found that maslinic acid and oleanolic acid inhibited DNA synthesis and activated apoptotic intrinsic pathways in human astrocytoma cancer cell lines. The apoptosis in brain cells triggered by triterpenic acids involves cytoskeletal and morphological mutations, the accumulation of intracellular ROS, loss of mitochondrial membrane integrity, and caspase‐3 activation (Tsai et al. 2023; Zhang et al. 2022).

Hydroxytyrosol has a remarkable neuroprotective effect on neuroblastoma cells in mice. The administration of hydroxytyrosol at doses of 10 and 50 mg/kg after an 8‐week duration significantly increased the activity of the mitochondrial respiratory chain and complex I in the brains of db/db mice. It also decreased the erythroid 2 nuclear factor, transcription factor, and factor 2, which include haem oxygenase, p62, protein oxidation, and enhanced superoxide dismutase. It activated AMP‐activated kinase, PPARγ coactivator, and sirtuin, which form an energy‐sensing platform of proteins known for regulating the response to oxidative stress and the function of mitochondria (Imran et al. 2018). Hydroxytyrosol (HT) exhibits significant neuroprotective effects, particularly on neuroblastoma cells. Research indicates that HT enhances mitochondrial function and reduces oxidative stress, which are critical for neuronal survival. In studies involving SH‐SY5Y neuroblastoma cells, HT administration improved cell viability and reduced markers of oxidative damage, suggesting its potential in neuroprotection against various stressors (Maiuolo et al. 2023; Zheng et al. 2015). Oleuropein at 10 μM in human glioblastoma cells (U87) reduced oxidative stress. Furthermore, the pretreatment of oleuropein systematically decreased the NO and inducible expression of NO synthase iNOS gene in these cells (Kucukgul et al. 2020).

In human glioblastoma cells, oleuropein and hydroxytyrosol protected against tumor necrosis factor‐induced cyclooxygenase expression. They suppress the expression of protein significantly. TNF induced ERK and JNK phosphorylation, PGE2 secretion (Imran et al. 2018). Autophagy is triggered through the Ca2+ axis by oleuropein in cultured cells. Specifically, it rapidly released Ca2+ from SR stores, which results in the activation of CAMKKβ, along with AMPK activation and phosphorylation. Furthermore, oleuropein decreases the immune reactivity of phospho‐ and mTOR‐phosphorylated substrate S6K levels, enhances (Rigacci et al. 2015). Previously, Lamy et al. (2016) determined that oleuropein and hydroxytyrosol have an inhibitory role against the expression of protein, tumor necrosis factor α‐induced cyclooxygenase expression, and the secretion of PGE2 in human glioblastoma cells. They also decreased TNF‐α‐induced ERK and JNK phosphorylation, as well as TNF‐α‐induced NF‐κB phosphorylation.

### 4.2 Esophageal Cancer (Upper Aerodigestive Tract)

Upper aerodigestive tract (UADT) consists of the larynx, esophagus, pharynx, facial skin, lower and upper jaw bones, paranasal and nose air sinuses, salivary glands, and oral cavity (Li, Doherty, and Wang 2022). The cancers of UADT are among the most common and prevalent cancers in humans. Therefore, it requires the development of a more effective prevention program (Fang et al. 2024; Reichenbach et al. 2019). Olive oil and its unsaturated fats have been negatively related to several cancer risks, particularly those of UADT (Bansal and Gupta 2022).

The most consistent evidence of olive oil's favorable role came from studies on the respiratory tract and upper digestive tract cancers. Regarding pharyngeal and oral cancer, high olive oil intake was associated with a significantly reduced cancer risk (Rodríguez‐Molinero et al. 2021). The eating patterns and food products are strongly linked to UADT progression, as margarine and mixed seed oils were not correlated with cancer risk, while a positive correlation appeared for butter. Patterns of fat intake exerted an impactful influence on the cancer risk of the pharynx and oral cavity than on colorectal and breast cancer. In a case–control study of esophageal cancer, the intake of olive oil was associated with a significant risk reduction, whereas consumption of butter was directly linked to the risk of esophageal cancer (Ruggiero et al. 2023). The analysis showed the association of seasoning fat with the risk of laryngeal cancer, and it was found that olive oil and other specific seed oils were more effective in reducing the risk of cancer development, while the oils from mixed seeds were directly associated with the risk of laryngeal cancer (Fresán et al. 2018).

### 4.3 Thyroid Cancer

Globally, the prevalence rate of thyroid cancer (TC) has increased over the last few decades. It is categorized in three types: medullary thyroid cancer, differentiated thyroid cancer (follicular and papillary), and undifferentiated (anaplastic and poorly differentiated) thyroid carcinoma (Chan 2023; Mishra 2022). Among these three, differentiated thyroid cancer accounts for 90% of cancers consisting of follicular and papillary thyroid carcinoma. Moreover, medullary TC (MTC) accounts for 5% of total thyroid cancers and occurs in parafollicular C cells (Prete et al. 2020). In 2023, it is estimated by the American Cancer Society that new proposed cases of thyroid cancer are 43,720 (31,180 females and 12,540 males), and 2120 died due to TC (1150 females and 970 males) (Mallick and Harmer 2024).

The antiproliferative effects of oleuropein and per‐acetylated oleuropein against TC were verified using BCPAP and TPC‐1 in comparison to noncancerous cell lines (i.e., TAD‐2). These compounds exhibited antiproliferative activities at concentrations below 100 μM (Gervasi and Pojero 2024). The decrease in Akt and ERK phosphorylation, which is important in cancer invasion and metastasis, was associated with the inhibition of DU145 and LNCaP prostate cell proliferation, leading to necrosis at oleuropein doses of 100 and 500 μM. Oleuropein reduced the oxidative stress by regulating the GSH (Guo et al. 2020). In another study, oleuropein doses (150–200 μM) in dose‐dependent manners obstructed the G‐1 and G‐2/M phases in human cancerous cells, enhanced the phosphorylation of Bax, cytochrome c protein, c‐Jun NH (2)‐terminal kinase (JNK) protein, p21, p53, and ATF‐2 in the cytoplasm. The effects of HT on follicular and (WRO) and papillary (FB‐2 and TPC‐1) cell lines were studied, which resulted in HT high concentrations decreasing the cancerous cell viability while simultaneously declining the expressions of cyclin D1 and improving regulation of p21 levels (cell cycle key modulator) (Gervasi and Pojero 2024; Toteda et al. 2017). According to Bulotta et al. (2013), oleuropein prominently inhibited the proliferation of two TC cell lines responsible for genotypic variations in papillary TC in humans. It was observed that oleuropein concomitantly decreased phospho‐ERK and basal phospho‐Akt levels, along with H_2_O_2_‐stimulated ROS rates.

### 4.4 Breast Cancer

Breast cancer is another major ailment among women, with an estimated 2.3 million by the end of 2020 worldwide. Breast cancer has been divided into several types, but invasive ductal carcinoma is the most common. Moreover, based on the mRNA gene expression level, breast cancer can be classified into molecular subtypes (Nolan et al. 2023; Łukasiewicz et al. 2021). Several amendable and unmodifiable risk factors are liable for breast cancer, such as gender, age, genetics, family history, smoking, physical activity (PA), reproductive history, body mass index (BMI), and dietary factors (Videnros et al. 2020; Eve et al. 2020). Obesity, lipoproteins, estrogens, and cholesterol are such aspects that have a significant role in breast cancer progression. In postmenopausal women, estrogens play a major role in adiposity development, thus prompting breast cancer. Early menstruation and menopause in elderly age result in steroid genesis in the gonads to normal breast progress and the development of breast malignancy (Pandrangi et al. 2022).

Nouri et al. (2020) suggested that oleuropein (OLP) triggers apoptosis due to the initiation of the p53 pathway, which is controlled by the *BCL2* and *BAX* genes. In addition, oleuropein limited the growth of tamoxifen‐resistant MCF‐7 cells; however, there was no change in regular breast epithelial cells. Messeha et al. (2020) reported that oleuropein altered the mRNA expression associated with the apoptosis process in two types of breast cancer cell lines, MDA‐MB‐231 and MDA‐MB‐468 and claimed that oleuropein is more efficacious in MDA‐MB‐468 than in MDA‐MB‐231. Oleuropein (OLP) decreases breast tumor cell progression by modulating the cell cycle, increasing p21 expression, and reducing NF‐κB and cyclin D1 expression (Choupani et al. 2019). Additionally, OLP prevents EMT through the downregulation of sirtuin1, which contributes to the restriction of breast cancer cell movement (Przychodzen et al. 2019). The role of olive oil bioactive components in the management of breast cancer is shown in Table 2.

**TABLE 2:** Role of olive oil bioactive components in management of breast cancer.

Olive oil and bioactive compounds	Cell lines/genes	Mechanism	References
Hydroxytyrosol	MCF‐7 cells	Inhibition of cell development through epidermal growth factor receptor (EGFR), lower expression of Ki‐67	El‐Azem et al. (2019)
Hydroxytyrosol	SKBR3 cells	Suppression of FAS, COX‐2 and Bcl‐2, promoted activity of FOXO3a and Nrf2	De Pablos et al. (2019)
Hydroxytyrosol	Hs578T MDA‐MB‐231 and BT549 breast cell lines	Reduction of MDA‐MB‐231 cell viability, suppression of 3‐MA and HGF, reversing LC3‐I	Lu et al. (2019)
Oleuropein	MCF‐7 cells and MDA‐MB‐231	Amplified apoptosis in MCF, augmented expression of TNFRS10B and decreased bcl‐2 expression	Asgharzade et al. (2020)
Oleuropein	Caspases (Casp1, Casp14), BNIP2 CYCS and GADD45A	Reduced HDAC4, apoptosis of MCF7, induction of apoptosis through increasing Bax protein and p53	Mansouri et al. (2019)
Oleuropein	Pro apoptotic genes (p53 and Bax), anti‐apoptotic Bcl‐2	Prohibited cell migration, reduced expression of HDAC2 and HDAC3	Bayat et al. (2019)
Oleuropein	Beclin‐1, and LC3‐II/LC3‐I, p63	Reduced expression of HER^2^ and MET receptors, downregulate expression of mTOR	Castejón et al. (2020)

### 4.5 Skin Cancer and Blood Cancer

The outermost skin layer is the stratum corneum, which is selectively permeable and has a depth of ∼approximately 10–20 μm, providing a shield against dehydration of the body by maintaining sufficient water within it (Yadav et al. 2019). Keratinocytes predominate in cells to form the epidermis, which has a thickness of 75 μm. The innermost layer is the dermis, which has a thickness of approximately 200 μm and is composed of fibroblasts, elastin, and collagen (Graham et al. 2019). Keratinocyte skin cancer (KSC) and cutaneous melanoma skin cancer (CMSC) are the most prevalent skin cancers that occur in individuals having white skin. However, the mortality rates due to cancers are low, but their prevalence rate is increasing globally. The primary aspect behind the CMSC and KSC is an increase in skin exposure to ultraviolet radiation (UVR) (Sharma et al. 2020). KSC generally involves two types of carcinomas, namely basal cell carcinoma (BCC) and squamous cell carcinoma (SCC), which have significantly higher prevalence rates compared to melanoma (Hasan et al. 2023). In specific conditions, contemporary therapies, such as Patidegib, BIL‐010 t, Sinecatechins, Photodynamic Therapy, Imiquimod, and 5‐FU (fluorouracil), are believed to be more effective in curing skin cancer. However, alginate bilayers consisting of Hydroxytyrosol (HT) are considered a latent alternative to topical chemotherapy for curing skin cancer (Ng and Tan 2015).

The utilization of oleacein and oleocanthal caused a decrease in viability and relocation of nonmelanoma skin cancerous cells (Polini et al. 2018). In the WST‐1 assay, oleocanthal demonstrated a prominent function against human melanoma cells compared with fibroblasts during cell viability trials. OL suppressed AKT and ERK1/2 phosphorylation, reducing Bcl‐2 expression and potentially improving skin cancer treatment (Fogli et al. 2016). Kugić et al. (2022) reported the highest concentration of oleacein in EVOO, which exhibits significant anticancer properties in A375 melanoma cells; however, it displays minimal toxic impacts on noncancerous keratocyte cell lines (HaCaT). Gu et al. (2017) observed the anticancer activities of OC, and the results showed that OC has the capability to prevent various cellular mechanisms in melanoma cells, including invasion, migration, and proliferation, and also induce apoptosis. OC downregulates the cancer‐linked genes, that is, MMP‐2, MMP‐9, Bcl‐xL, and Mcl‐1, inhibits the signal transducer and activator of transcription 3 (STAT3) phosphorylation and its initiation. Moreover, it also involves the suppression of Src kinases and JAK2 expression, resulting in the inhibition of cell assault and angiogenesis. According to Emma et al. (2021), OC inhibited the AKT and ERK1/2 phosphorylation and downregulated the expressions of Bcl‐2, which were responsible for obstructing the cell propagation in melanoma cells in comparison with dermal fibroblasts.

Leukemia is a distortion of neoplastic cells that invade the blood and hematopoietic organs and consequently infiltrate various other tissues (Imran et al. 2018; Wang et al. 2024). The most identified blood cancers include non‐Hodgkin lymphoma, multiple myeloma, and acute lymphoblastic, acute myeloid, and chronic lymphocytic leukemia. Studies have shown that OOPE is capable of alleviating oxidative damage to DNA in leukemia cells of the HL60 promyelocytic line (Barbar and Sathick 2021). Moreover, OC inhibited Hsp90 due to a significant decrease in the expression of Hsp90 proteins (Cdk4 and AKT) in U937 cells associated with histiocytic lymphoma. The ATPase activity inhibition by the chaperone was observed due to a noncovalent linkage between the OC and Hsp90‐ATP binding sites. OC obstructs the cell cycle in the G2/M phase and initiates the apoptosis of U937 cells (Fogli et al. 2016).

### 4.6 Stomach Cancer

Stomach cancer, referred to as gastric cancer, is a significant menace to health and one of the major causes of cancer‐associated mortalities around the globe (Singh 2024). According to stats, approximately 1.1 million cases were reported in 2020, thus making it 4th in cancer morbidities and 7.7% deaths, ranking it 3rd among cancer fatalities (Ilic and Ilic 2022). Multiple factors are accountable for stomach cancer, such as social factors (smoking, alcohol, obesity, and low physical activity), dietary factors (high intake of smoked and salty food), and environmental factors (heavy metals and chemical exposure). In addition, pathogenic bacteria, such as *Helicobacter pylori* ( *H. pylori* ), are also responsible for malnutrition and the progression of disease (Collatuzzo et al. 2021). The pathophysiology revealed that the claudin‐6 (CLDN6) and ATAD2 genes are involved in the development of malignancy. The expression of CLDN6 is reduced in gastric cancer associated with pathological stage; however, ATAD2 expression is high due to Hypoxia‐inducible factor 1‐alpha (HIF1α) (Nayak et al. 2020). Numerous therapies are used to treat or manage stomach cancer, including gastrectomy, lymphadenectomy, preoperative chemotherapy, preoperative radiation therapy, postoperative adjuvant chemotherapy, and immunotherapy (Li, Doherty, and Wang 2022).

Olive oil dietary therapy for managing gastric carcinoma is a valuable approach, and research on olive oil and its phenolic components has demonstrated significant improvements in reducing cell insurgence (Zheng et al. 2022). The RNA nucleoside (8‐OH‐dG) is a significant biomarker of oxidative stress‐associated DNA damage, resulting in elevated superoxide radical. This cytotoxic mediator interacts with DNA synthesis and generates DNA cross‐linking, which regulates cell cycle development and, ultimately, contributes to the development of gastric cancer (Rybak et al. 2019). Maiuolo et al. (2021) investigated cisplatin (CIS)‐induced gastro‐ and lung cancer to explore the potential of oleuropein (OLE), a phenolic compound found in olive oil. At the completion of the study, they observed a significant drop in malondialdehyde (MDA) levels, total antioxidant status (TAS), and 8‐OH‐dG when rats were injected with a single dose (7 mg/kg) of CIS and treated with 50, 100, and 200 mg/kg OLE for three consecutive days. The effect of oleuropein on gastric carcinoma was investigated, resulting in a significant decline in cell proliferation, which supports the anticancer properties of olive oil (Castejón et al. 2020; Barzegar et al. 2019).

### 4.7 Liver Cancer

Liver cancer is another major threat to humans and ranked 6th among cancers, and hepatocellular carcinoma (HCC) is the major type among liver cancers. Since 2020, the worldwide burden of liver cancer has tripled, and the stats demonstrate that almost 905,700 people were identified and 830,200 people died from HCC. The maximum prevalence rate was reported in Eastern Asia, followed by Northern Africa (Li, Doherty, and Wang 2022; Okeke et al. 2020). Several risk factors contribute to the incidence of liver cancer, such as obesity, gender, Type 2 diabetes, race, smoking, chronic viral hepatitis, alcohol drinking, cirrhosis, inherited metabolic diseases, and exposure to carcinogens like aflatoxins. The occurrence can be reduced through preventive measures and modification in risk factors (Huang et al. 2021). Liver cirrhosis is one of the major causes of liver cancer as repetitive inflammation and fibrosis predispose the liver to neoplasm and malignancy. Hepatocellular carcinoma (HCC) originates from stem cells, and alpha‐fetoprotein (AFP) is a key biomarker for HCC. Alpha‐fetoprotein (AFP) is involved in the cell growth, differentiation, and apoptosis; moreover, people with HCC indicate elevated levels of AFP and a declining number of dendritic cells result in decreased interleukin 12 (IL‐12), which is tangled in the activation of receptors on natural killer (NK) cells known for killing of neoplastic cells (Fox et al. 2022; Geong et al. 2019).

**FIGURE 2:**
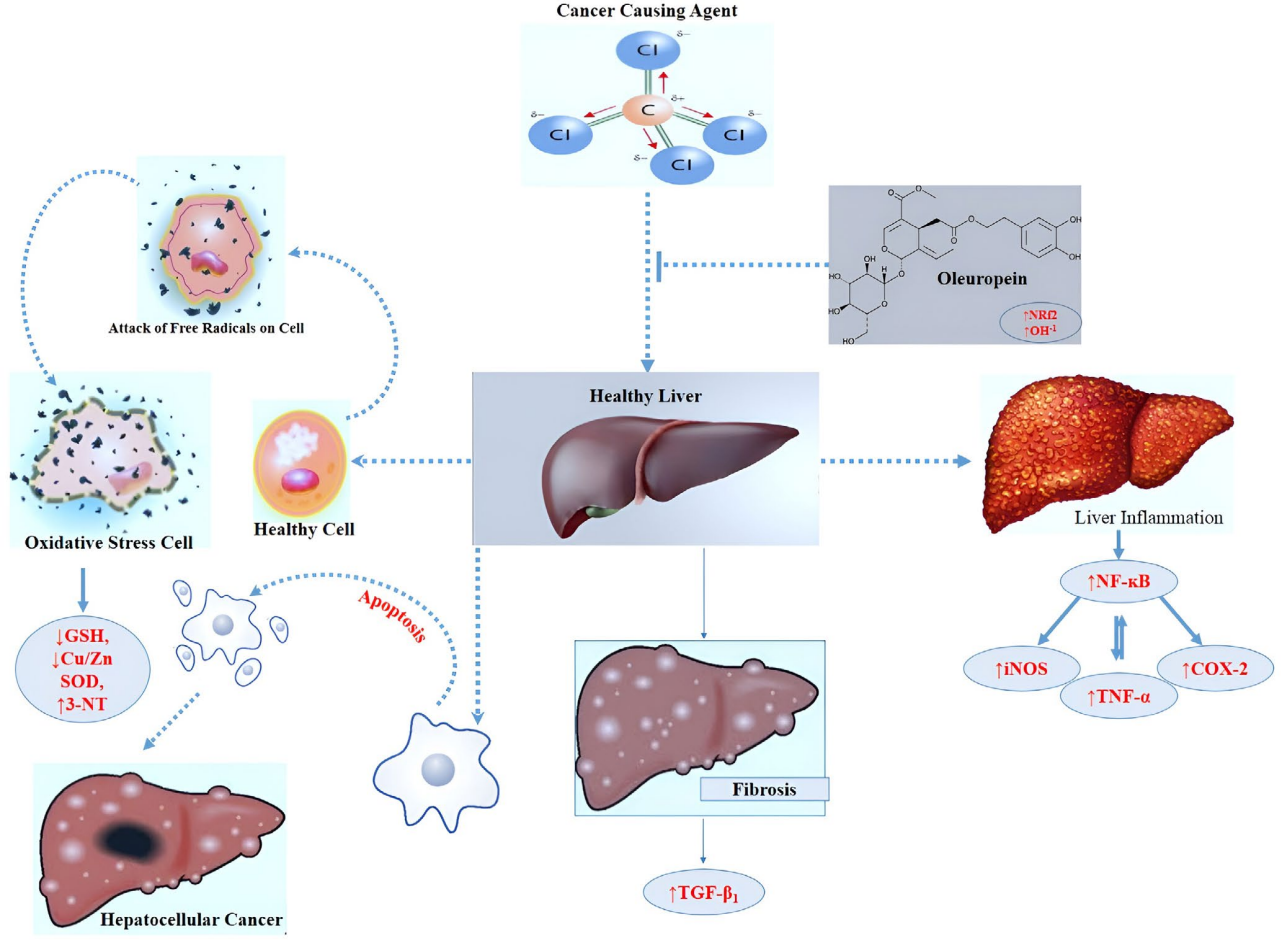
Preventive role of oleuropein against of liver cancer.

The polyphenols in olive oil have the potential to reduce tumor cells, as research on extra‐virgin olive oil (EVOO) polyphenols against HCC cell lines was conducted by De Stefanis et al. (2019) and reported a reduction in the propagation of three liver cancer cell lines (Hep3B, HepG2, and Huh7). They concluded that this effect is due to autophagy potentiated by tumor necrosis factor α (TNF‐α). Several pathways are responsible for cell death, and some of them are involved in protein metabolism. One of the pathways is the Akt/mTOR pathway, which is intricately involved in the modulation of anabolic signaling and protein production. Furthermore, this pathway is typically associated with the downregulation of autophagy. When cell lines HepG2 and Huh7 were exposed to EVOO extract, no downregulation was observed in the Akt–mTOR–p70 pathway (Emma et al. 2021). Furthermore, polyphenols function as free radical scavengers, regulating the Nrf2‐dependent antioxidant signaling pathway, thus defending cells from oxidative stress and apoptosis. Extra‐virgin olive oil (EVOO) extract has the ability to reduce the release of reactive oxygen species (ROS) in both HepG2 and Huh7 cells (Castejón et al. 2020). The preventive role of oleuropein against liver cancer is shown in Figure 2.

### 4.8 Lung Cancer

Lung cancer, also known as lung adenocarcinoma, covers a diverse range of types, that is, nonsmall cell carcinoma, small cell carcinoma, large cell carcinoma, and pulmonary blastoma. The disease burden reveals almost 2.2 million cases reported in 2020, which makes it the second most common cancer worldwide (Duma et al. 2019). Smoking and arsenic‐contaminated water are the main causes of lung cancer; moreover, high intake of ꞵ‐carotene supplement can also increase the risk of lung cancer. Moreover, these factors, including excessive consumption of processed meat or red meat and alcohol consumption, also play a role in the occurrence (Rudin et al. 2021). Exosomes are one of the major biomarkers in lung cancer, which are nano vesicles linked with multiple pathological and physiological conditions. These components are released from various cells, including immune and cancer cells, and these particles have the ability to alter the function of cells through the intercellular transmission of their DNA, proteins, mRNA, and microRNAs (Amiri et al. 2021). The main root of mortality in lung cancer is metastasis, and bone marrow‐derived mesenchymal stem cells (BMSCs) are such cells that contribute to tumor progression. Research on microRNAs in exosomes has revealed that they can promote cell division and induce metastasis of lung adenocarcinoma by activating STAT3 signaling‐induced epithelial‐to‐mesenchymal transition (EMT) (Zhang et al. 2019).

Hydroxytyrosol, a polyphenol found in olive oil, has been shown to be effective in reducing the proliferation of lung cancer cells. Gallazzi et al. (2020) investigated the use of hydroxytyrosol in treating lung cancer and reported significant efficacy of this compound. This polyphenolic component has the ability to decrease the expression of CXCR4 and CXCL12 receptors, as well as STAT3 phosphorylation, in lung cancer. Furthermore, it inhibits adhesion capabilities on the fibronectin layer and reduces the ability to produce invasive sprouts in the Matrigel layer. Oleuropein is another phenolic compound found in olive oil, which is associated with antineoplastic properties, as it has been shown to be efficacious against a wide range of tumors, including gastric, ovarian, breast, and pancreatic (Zheng et al. 2022). Bossio et al. (2022) investigated apoptosis in A549 cells induced by oleuropein, which upregulates mitochondrial Glo2 (mGlo2), thereby facilitating the superoxide anion and Akt signaling pathways. Additionally, oleuropein has the ability to reduce oxidative stress by enhancing mitochondrial function through the initiation of the Nrf2‐mediated signaling pathway; thus, in this manner, it can prevent hypothalamic damage (Pacifico et al. 2022).

### 4.9 Colorectal Cancer

According to WHO, colorectal cancer (CRC) is the third leading cancer and ranks second in fatalities worldwide. The data for 2024 provided by the American Cancer Society (ACS) confirmed that 106,590 new cases of colon cancer and 46,220 new cases of rectal cancer were reported in the US. Colon and ileal mucosa consist of diamine oxidase (DAO) enzyme in higher concentrations and are capable of breaking down histamines, while bile salts are capable of preventing the DAO, resulting in an increase in sequences of carcinoma and mucosal propagation. Oleuropein and hydroxytyrosol can affect the patterns of secondary bile in the colon to stimulate the polyamine metabolism mechanism, which can reduce the proliferation of normal mucosal cells into carcinoma cells (Imran et al. 2018). OC demonstrated significant anticancer activity against colorectal carcinoma (CRC) cells. It has the capability to stimulate γH2AX (a DNA‐damaging marker) expression, which activates mitochondrial depolarization and enhances ROS production. Furthermore, the ROS scavenging compound (N‐acetyl‐L‐cysteine) was also suppressed by OC (Cusimano et al. 2017). Virgin olive oil utilization was shown to exhibit apoptotic and antiproliferative properties in HT‐29 and Caco‐2 cell lines, which are associated with cancer, as well as major catabolites, including catechol, dihydroxy phenyl propionic acid, hydroxyphenylpropionic acid, phenylpropionic acid, and phenylacetic acid, which are present in the feces of individuals. Hydroxyphenylpropionic acid, phenylacetic acid, and hydroxytyrosol cause obstruction in the cell cycle and boost apoptosis. Moreover, Caco‐2 cell lines were observed to be less sensitive to OC treatment in comparison with HT‐29 (Lopez de las Hazas et al. 2017).

Extra‐virgin OOPE increased the expression of estrogen receptor (ER) β, resulting in the inhibition of cell proliferation in colon cancerous cells and the initiation of receptors similar to those activated by 17β‐estradiol 9 receptor (Pampaloni et al. 2014). Moreover, HTyr was shown to reduce the levels of epidermal growth factor receptor (EGFR) in HT‐29 xenografts and colorectal carcinoma cells. EGFR plays a crucial role in various stages of cancer development, including invasion, proliferation, angiogenesis, and apoptosis. EGFR is activated by its rapid degradation through both proteasomal and lysosomal mechanisms. HTyr was depicted as having the potential to increase the degradation rate of EGFR in cancerous cells, with no impact on the levels of EGFR in healthy colon cells (Van Rymenant et al. 2018). Terzuoli et al. (2016) reported that induced ubiquitination results in the degradation of EGFR in lysosomes both in vivo and in vitro, a molecular mechanism that can be utilized to evaluate the antiproliferative impact of HTyr therapy in CRC. Similarly, oleuropein in the basal diet of rats stimulated by azoxymethane (AOM) provoked colon cancer and decreased DNA damage in marginal leukocytes. Moreover, it significantly decreased the AOM‐stimulated cancer prevalence from 57% to 14% in colon segments (Sepporta et al. 2016). Likewise, the administration of oleuropein in mice, combined with both AOM and dextran sulfate sodium (DSS), stimulated a decline in inflammatory markers and a decrease in colon cancer development (Giner et al. 2016). Terzuoli et al. (2017) reported the potential of HT in olive oil against cancer during cetuximab chemotherapy. HT enhanced the effects of EGFR inhibitors, which play a key role in the treatment of colon cancer patients. HT acetates also depicted the upregulation of xenobiotic‐metabolizing enzymes CYP1A1 and UGT1A10, and an improvement in carcinogen detoxification. Additionally, HT is also well known for its inhibition of cancer propagation, stimulating cell cycle arrest and apoptosis in various cancers, such as cholangiocarcinoma (Li et al. 2014).

The concentrations of OC (25–50 μM) stimulated apoptosis and prevented colony development in SW480 and HT29 colon cancer cells by enhancing ROS formation, which can cause DNA damage leading to cell death (Cusimano et al. 2017). Hydroxytyrosol promoted ROS production, which activated the PI3K/AKT/FOXO3 pathway with subsequent regulation of FOXO3 targets, including catalase and SOD, resulting in the induction of apoptosis in DLD1 colon cancer cells and a reduction in cellular antioxidant defenses (Sun et al. 2014). Storniolo et al. (2019) explored that oleic acid (1–100 μM) stimulated the DNA formation and growth of Caco‐2 cells (two times greater in comparison with cells without growth factor). Oleuropein and Hydroxytyrosol (0.1–10 μM) reverse the Caco‐2 cell growth and DNA formation stimulated by oleic acid. HT regulates the arachidonic acid cascade, which is linked to its antitumor activity. Torić et al. (2020) studied the biological impact of extra‐virgin olive oil phenolic extracts (EVOO‐PEs) on colon (SW48) and cervical (HeLa) cancer cell lines in humans separately and in combination with irinotecan, 5‐fluorouracil (5‐FU), carboplatin (CBP), and cisplatin (cDDP). The findings suggested that EVOO‐PEs of different varieties (Žižolera, Buža, and Oblica‐Sea) along with anticancer medicines enhanced the metabolic activities of SW48 and HeLa cell lines, depicting a protective role. Di Francesco et al. (2015) explored the effect of EVOO, along with its phenolic constituents, on the modulation of the endocannabinoid system (ECS) gene expression via an epigenetic mechanism in Caco‐2 colon cancer cells in humans. HTyr and oleuropein contributed to the restoration of CB1 gene expression by reducing the methylation levels of gene promoters, resulting in a decrease in the propagation of cancerous cells during an in vitro study.

### 4.10 Ovarian Cancer

The incidence of ovarian cancer (OC) in females is increasing every day. According to statistics, 313,959 new cases of ovarian cancer were reported in 2020, thus making it 3rd in rank after cervical and uterine cancer. The 2% population is at risk of developing ovarian cancer; in other words, 2 out of 100 women are at risk of developing this cancer (Hack et al. 2020; Lheureux et al. 2019). Studies have shown that 90% of OC is epithelial, while 10% is nonepithelial. Among epithelial ovarian cancer, 3% is mucinous, whereas others are nonmucinous. Nonmucinous are further classified into serous (70%), clear cell (10%), and endometrioid (10%) (Momenimovahed et al. 2019). A diverse range of factors is considered to initiate ovarian carcinoma, such as age, menopause, parity, pregnancy characteristics, pelvic inflammatory disease (PID), ovarian cysts, genetics, hormonal, and dietary factors (Stewart et al. 2019). Oxidative stress is a major cause of progression and triggering of neoplasm; moreover, oxidative stress can trigger further transcription factors, including nuclear factor erythroid 2‐related factor 2, nuclear factor (NF)‐κB, hypoxia‐inducible factor 1 (HIF‐1), and peroxisome proliferator‐activated receptor γ. The factors can regulate the expression of many genes responsible for cellular mechanisms such as inflammation, cell proliferation, apoptosis, and differentiation (Tossetta and Inversetti 2023; Evans et al. 2023).

Sheikhshabani et al. (2021) investigated the effects of oleuropein on apoptosis, cell viability, cisplatin resistance, and the expression levels of miR‐16, miR‐34a, miR‐21, miR‐125b, and other target genes in ovarian cancer cells. The findings revealed that the manifestation of P21, TNFRSF10B, and P53 increased, whereas the manifestation of Bcl‐2 and Mcl‐1 declined. Furthermore, a significant decline in the manifestation of miR‐21 and an upsurge in the expression of miR‐34a, miR‐16, and miR‐125b were observed with oleuropein. Benot‐Dominguez et al. (2021) stated that olive oil (OO) decreases cell proliferation by affecting the cell cycle and promotes apoptosis through mitochondrial damage, which leads to a reduction in tumor progression. Ly et al. (2021) investigated oleuropein and reported that it regulates miRNA expression, thus resulting in decreased cisplatin resistance, initiates apoptosis, and inhibits cell proliferation. In addition, OLP promotes sensitivity to radiotherapy in ovarian tumor patients.

Similarly, studies have proven that EVOO and its polyphenols have the potential to ameliorate bladder cancer. Previously, Coccia et al. (2016) reported that the ability of polyphenols from EVOO has the potential to inhibit the growth of bladder cancer. In a dose‐dependent manner, the results showed that EVOO significantly inhibited the growth and proliferation of cancer cells. Additionally, the cell cycle after EVOO treatment showed that growth stopped in the G2/M phase before mitosis and EVOO promoted apoptosis. Xing et al. (2017) demonstrated that radon treatment of ovary cells rendered sensitive to oleuropein altered the microRNA expression profile. Endogenous expression of miR‐299 was turned down by a hypoxia‐inducible factor and turned back up by oleuropein. This downregulation of HPSE1 made cells more sensitive to radiation. Scicchitano et al. (2023) also confirmed that high amounts of oleuropein inhibit the growth of HEY and MCF‐7 cells, leading to their death. Their results also show that low doses of oleuropein lower the levels of LIP and ROS in ovarian cancer patients who are being treated with erastin.

Elevated expression of PAI‐1 has been correlated to adverse results in a number of human cancers, including breast, stomach, and ovarian cancers. Tzekaki et al. (2021) found that elevated PAI‐1 levels are negatively related to PR and ER expressions in a large group of human cancers that respond to estrogen and progesterone. They demonstrate that EVOO or oleuropein treatment acts as a natural PAI‐1 inhibitor, causing PAI‐1 levels to become less stable in ER‐/PR‐ cancer cells only. This is followed by caspase activation and a slowing of cell growth. Likewise, Coccia et al. (2014) showed that olive oil polyphenol (OOPE) has the potential to prevent bladder cancer cells from expanding and invading, possibly by reducing matrix metalloproteinase 2 (MMP2).

### 4.11 Prostate Cancer

Prostate cancer (PC) ranks among the most prevalent forms of cancer, and in addition to genomic mutations and familial predisposition, environmental lifestyle variables have been suggested as potential contributors to the development and advancement of prostate cancer. Moreover, there is a potential association between metabolic syndrome and an elevated risk of prostate cancer (Pandareesh et al. 2021). The mortality rate of PC may be elevated by cigarette smoking and obesity, whereas consistent physical activity can mitigate the progression of the disease. Furthermore, higher consumption of fried fat, dairy products, and processed meat is associated with the incidence of prostate cancer (Chung et al. 2019).

Several in vitro studies have demonstrated that phenolic components derived from olive oil exhibit anticancer activities against various types of cancer, in relation to the cancer illnesses and phenolic compounds found in EVOO (Siddique et al. 2019). In a study, OLE was dosed at 75 mg/kg for three times/week in a xenograft model of nude mice to investigate its anticancer impact against PC. Moreover, PC cell lines (LNCaP, DU‐145, PC‐3) were also treated with OLE. The findings showed that 1‐ and 2‐mM OLE reduced cell migration by 15% and 69%, respectively. Additionally, in mice, OLE supplementation downregulated the expression of PCSK1, PCSK2, and PCSK9 (Ahmed et al. 2025). Research indicates that the phenolic substances in EVOO have the potential to inhibit oncogenic factors, such as mutations, the catalytic functions of key metabolic and genetic pathways, and interactions that affect DNA methylation (Verdura et al. 2019). The results of another study suggest that the hydroxytyrosol‐rich extract made from the effluent of an olive mill efficiently prevents the growth of prostate cancer. The findings showed that cell growth, migration, invasion, and adhesion are all suppressed due to extract supplementation (Rodríguez‐López et al. 2020).

Goren et al. (2019) revealed that extra‐virgin olive oils naturally abundant in oleocanthal exhibited a significant decrease in the viability of cancer cells and triggered lysosomal membrane permeabilization. Conversely, oils that were deficient in oleocanthal did not exhibit these effects. Furthermore, it was discovered that the administration of oleocanthal resulted in an increase in the lifespan of mice that were genetically modified to develop pancreatic neuroendocrine tumors. In a recent study by Siddique et al. (2022), it was found that S‐(−)‐oleocanthal (OC), an ingredient of the EVOO, showed inhibitory effects on SMYD2 and reduced colony formation, invasion, and migration of several PC cell lines, including PC‐3M, PC‐3, CWR‐R1c, and DU‐145. The expansion of mCRPC in CWR‐R1ca cells that were engrafted into male nude mice was significantly reduced by the daily oral administration of OC at a dose of 10 mg/kg for a period of 11 days. Moreover, serum prostate‐specific antigen (PSA) levels in mice given OC therapy significantly decreased.

According to Antoniou and Hull's (2021) findings, the viability of human prostate cancer cells (DU145, PC‐3) is reduced by hydroxytyrosol. According to a study conducted by Imran et al. (2018), the introduction of hydroxytyrosol at a concentration of 80 μmol/L to PC‐3 cells resulted in an increase in superoxide concentration and the initiation of apoptotic cell death. The aforementioned alterations have been linked to the process of autophagy, impairment of mitochondrial function, and activation of MAP kinases. The study demonstrates that the generation of superoxide by hydroxytyrosol leads to mitochondrial dysfunction and apoptotic cell death in PC‐3 cells. Another study demonstrated that the coadministration of oleuropein and hydroxytyrosol has the potential to halt the progression of the cell cycle, elevate the Bax/Bcl‐2 ratio, enhance the activation of caspase‐3/7, and induce apoptosis in MIA PaCa‐2 cells, a type of pancreatic cancer cell. Moreover, there was an elevation in the expression of JUN and FOS (protooncogenes) within MIA PaCa‐2 cells. The dimerization of C‐JUN and C‐FOS to AP‐1 has been suggested as a possible mechanism for inducing apoptosis in MIA PaCa‐2 cells (Goldsmith et al. 2018).

## 5 Limitations and Future Perspective

Olive oil and its polyphenolic constituents, particularly oleuropein, have shown anticancer activity in cell cultures and animal models. However, bioavailability and metabolism could be significant limitations hindering effectiveness. Orally ingested polyphenols undergo first‐pass metabolism in the gut and liver, producing conjugates whose anticancer potency may be reduced compared with the parent compounds. Moreover, the dose and route may also limit the anticancer impact. Administering pharmacologically relevant doses may require supplementation beyond normal culinary use, which raises concerns about safety and tolerability. The major issue is the lack of evidence‐based clinical trials. The existing trials are short‐term and lack results, such as patient survival and tumor reduction. The polyphenol content fluctuates widely depending on olive cultivar, harvest time, processing methods, and storage conditions, making the standardization of therapeutic formulations challenging. Future investigations are likely to focus on enhancing bioavailability through the use of nano‐formulations, targeted delivery systems, and combinations with existing therapies. Advances in precision medicine may enable patient‐specific polyphenol regimens, leveraging genetic and metabolic profiling to maximize efficacy. Clinical trials are crucial for validating preclinical findings and establishing standardized dosage guidelines.

## 6 Conclusion

Cancer or neoplasm is a state in which cells from any body organ start to multiply abnormally or become out of control. The general mechanism of cancer involves the activation of oncogenes, inactivation of tumor suppressor genes, and modifications of signaling pathways. Numerous preventive and curative approaches are employed to treat cancer, and dietary therapy is particularly valuable and efficacious due to its availability, cost‐effectiveness, and safety. Several naturally occurring foods have been shown to be beneficial in cancer management. Olive oil (OO) and its bioactive compounds are highly admired for reducing inflammation and cell insurgence. Multiple studies have proven that olive oil and its bioactive components have the potential to regulate gene expression and modulate pathways involved in cell proliferation. However, despite growing evidence on the anticancer effects of olive oil polyphenols, particularly hydroxytyrosol and oleocanthal, the precise molecular mechanisms underlying their effects remain poorly understood. Most current studies are preclinical, with limited clinical trials confirming efficacy in humans. There is also a lack of standardization in polyphenol dosage, formulation, and delivery methods, leading to inconsistent results across studies.

## Author Contributions

**Muhammad Junaid Anwar:** conceptualization (equal), writing – original draft (equal). **Muhammad Hammad Anwar:** conceptualization (equal), writing – original draft (equal). **Muhammad Imran:** conceptualization (equal), writing – original draft (equal). **Ahmad Mujtaba Noman:** writing – original draft (equal). **Muzzamal Hussain:** data curation (equal), investigation (equal), supervision (equal). **Hassan Raza:** investigation (equal), validation (equal), visualization (equal). **Hagar M. Mohamed:** investigation (equal), writing – review and editing (equal). **Gamal A. Mohamed:** data curation (equal), writing – review and editing (equal). **Sabrin R. M. Ibrahim:** data curation (equal), investigation (equal), supervision (equal). **Tadesse Fenta Yehuala:** supervision (equal), validation (equal), visualization (equal), writing – review and editing (equal). **Suliman A. Alsagaby:** data curation (equal), investigation (equal), resources (equal). **Waleed Al Abdulmonem:** investigation (equal), methodology (equal), resources (equal). **Mohamed A. Abdelgawad:** writing – original draft (equal). **Ehab M. Mostafa:** investigation (equal), validation (equal), visualization (equal). **Mohammed M. Ghoneim:** conceptualization (equal), investigation (equal). **Samy Selim:** data curation (equal), investigation (equal).

## Conflicts of Interest

The authors declare no conflicts of interest.

## Data Availability

The data that support the findings of this study are available from the corresponding author upon reasonable request.
